# Low temperature lignocellulose pretreatment: effects and interactions of pretreatment pH are critical for maximizing enzymatic monosaccharide yields from wheat straw

**DOI:** 10.1186/1754-6834-4-11

**Published:** 2011-05-13

**Authors:** Mads Pedersen, Katja S Johansen, Anne S Meyer

**Affiliations:** 1 Bioprocess Engineering, Department of Chemical and Biochemical Engineering, Building 229, Technical University of Denmark, DK-2800 Kgs, Lyngby, Denmark; 2 Novozymes A/S, Krogshøjvej 36, DK-2880, Bagsværd, Denmark

## Abstract

**Background:**

The recent development of improved enzymes and pentose-using yeast for cellulosic ethanol processes calls for new attention to the lignocellulose pretreatment step. This study assessed the influence of pretreatment pH, temperature, and time, and their interactions on the enzymatic glucose and xylose yields from mildly pretreated wheat straw in multivariate experimental designs of acid and alkaline pretreatments.

**Results:**

The pretreatment pH was the most significant factor affecting both the enzymatic glucose and xylose yields after mild thermal pretreatments at maximum 140°C for 10 min. The maximal enzymatic glucose and xylose yields from the solid, pretreated wheat straw fraction were obtained after pretreatments at the most extreme pH values (pH 1 or pH 13) at the maximum pretreatment temperature of 140°C. Surface response models revealed significantly correlating interactions of the pretreatment pH and temperature on the enzymatic liberation of both glucose and xylose from pretreated, solid wheat straw. The influence of temperature was most pronounced with the acidic pretreatments, but the highest enzymatic monosaccharide yields were obtained after alkaline pretreatments. Alkaline pretreatments also solubilized most of the lignin.

**Conclusions:**

Pretreatment pH exerted significant effects and factor interactions on the enzymatic glucose and xylose releases. Quite extreme pH values were necessary with mild thermal pretreatment strategies (T ≤ 140°C, time ≤ 10 min). Alkaline pretreatments generally induced higher enzymatic glucose and xylose release and did so at lower pretreatment temperatures than required with acidic pretreatments.

## Background

With the ambitious targets set in both the US and Europe for increasing the share of renewable fuels in the transport sector, ethanol production from cellulosic biomass is currently receiving significant attention as a renewable, environmentally friendly alternative to fossil fuels [1,2]. One of the first prerequisites in such ethanol production is the efficient generation of a fermentable hydrolysate from the biomass feedstock. Significant progress has recently been made with respect to (a) development of better enzymes for catalyzing the degradation of cellulose and hemicellulose (mainly xylan) to monosaccharides [3], and (b) engineering of inhibitor tolerant pentose fermenting yeast strains and relevant fermentation regimes for efficient cofermentation of glucose and xylose to ethanol [4,5]. This progress now calls for improvement of the biomass pretreatment step both because attention is needed to both glucose and xylose utilization, and because of the very high temperatures widely used for pretreatment, which is one of the main processing steps limiting the cost effectiveness of converting cellulosic biomass to ethanol [1,2,6].

It is well recognized that lignocellulosic substrates have to be subjected to a hydrothermal or thermochemical pretreatment prior to the enzymatic hydrolysis. The purpose of the pretreatment step is mainly to increase the responsivity of the cellulose to enzymatic attack, but the pretreatment now also needs to prepare for optimal utilization of the hemicellulose, currently mainly the xylose released from the hemicellulose [1,4,5,7,8]. Hence, an effective pretreatment must achieve good hemicellulose conversion yields and produce an easily digestible cellulosic solid. Currently, the most studied types of pretreatments include dilute acid thermochemical pretreatment (with or without steam explosion), steam or liquid hot water treatments, ammonia fiber expansion (AFEX), and alkaline wet oxidation or wet explosion treatments [7-14]. A few pretreatment methods such as lime (calcium hydroxide) and the so-called COSLIF (cellulose solvent (concentrated phosphoric acid) and organic solvent (95% ethanol)) treatment [15] are conducted at relatively low temperatures, but then have the disadvantage of requiring high addition loads of lime or solvent, respectively, and very long treatment times ranging from 60 min to several hours. The other pretreatment procedures mentioned above vary with respect to reaction conditions and catalytic mechanism, but are all energy-demanding, thermal procedures mostly involving treatment at temperatures above approximately 160°C to 180°C [7,14]. It is well known, and has been convincingly shown for both corn stover and wheat straw, that the pH during pretreatment has a significant influence on the lignin and hemicellulose solubilization, and in turn on the subsequent enzyme catalyzed hydrolysis of the lignocellulosic substrates [14,16,17]. Generally, with acid-catalyzed pretreatment regimes, a significant amount of the biomass hemicellulose fraction is hydrolyzed during the actual pretreatment, that is, directly solubilizing the C5 saccharides including xylose [14]. In contrast, the alkali-based pretreatment methods are more effective at solubilizing the lignin [14,17,18]. However, the quantitative interactions among different pretreatment parameters, that is, the pH, treatment temperature, and time, for obtaining both maximal enzymatic glucose and xylose yields are not clear.

In Europe most research on second-generation ethanol production, including pretreatment and enzymatic saccharification, is focused on straw, notably wheat straw. This substrate is also gaining increased attention in the US as a cellulosic ethanol feedstock [1,8,9,13,18-20]. Hence the purpose of this work was to examine and evaluate the influence of pretreatment pH and the possible interactions between pH and temperature during pretreatment on the enzymatic xylose and glucose yields, and to unravel any possible correlations between the enzymatic xylose and glucose liberation and the pretreatment conditions. The effects of pretreatment pH, temperature, and holding time on the subsequent enzymatic xylose and glucose yields were evaluated in two central composite experimental design templates: one for low pH (pH 1 to 4) and one for high pH (pH 10-13), at varying temperatures (100°C to 140°C) and holding times (0-10 min). Each template included 15 different experimental combinations of the factors with 3 replicated center points. An additional objective was to examine if any of the mild thermal pretreatment strategies could solubilize lignin while leaving the remaining straw biomass digestible for enzymatic hydrolysis and high monosaccharide yield recoveries. Pretreatments were carried out in a custom-built laboratory-scale loop pump reactor and the enzyme catalyzed monosaccharide liberation was accomplished via use of a low-dosage benchmark enzymatic treatment using a novel, commercial cellulolytic enzyme preparation, Cellic C-Tec (Novozymes A/S, Bagsværd, Denmark).

## Results and discussion

### Enzymatic glucose and xylose release from the solid wheat straw fraction after pretreatment

**Figure 1:**
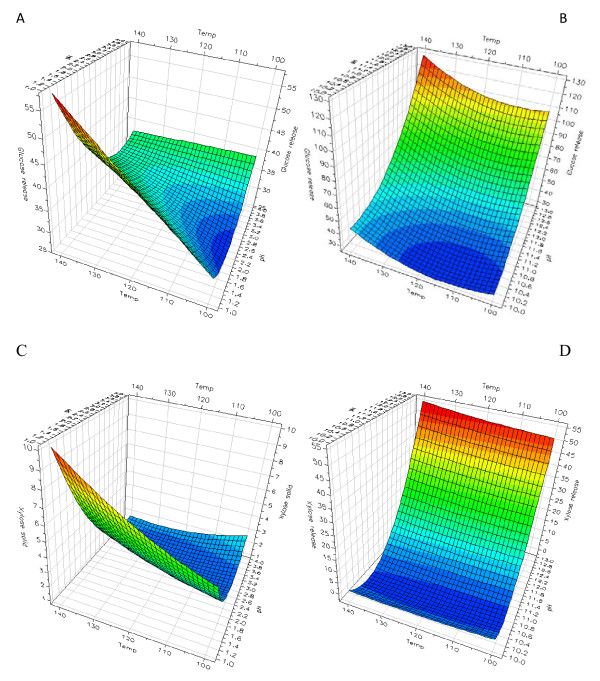
**Enzymatic glucose and xylose release from pretreated solid fractions**. Response surface modeling of enzymatic glucose release from wheat straw pretreated at **(a) **acidic and **(b) **alkaline conditions, and enzymatic xylose release from wheat straw pretreated at **(c) **acidic and **(d) **alkaline conditions. Temperature is in°C and monosaccharide releases in g/kg original dry matter. All samples were pretreated for 10 min.

In the acidic pH range (pH 1-4), the pretreatment pH alone did not affect the subsequent enzymatic glucose and xylose release from the solid wheat straw fraction markedly (Table 1, Figure 1a, c). Despite the lack of a neat effect of the pH at acidic pretreatments the multivariate data analysis revealed a significant interaction effect of the pretreatment pH and temperature, signifying that the higher the pretreatment temperature and the lower the pH in acidic pretreatments the higher the enzymatic monosaccharide release from the pretreated solid wheat straw. The pretreatment temperature itself also clearly had a significant influence on the enzyme catalyzed monosaccharide release from pretreated solid wheat fractions pretreated at acidic conditions, that is, the higher the pretreatment temperature, the higher the subsequent enzyme catalyzed monosaccharide release (Table 1, Figure 1a, c). It was evident that this effect was most pronounced at the lowest pH values, corroborated by the significant pH·T interaction (Table 1, Figure 1a, c). As will be discussed later, the enzyme catalyzed xylose release from the acid pretreated solid wheat fraction did not increase significantly when the enzyme dosage was increased mainly because of the high solubilization of xylose directly during acidic pretreatment (Table 2). Regarding alkaline pretreatments, pH had a clear positive effect on enzymatic glucose and xylose release (that is, the higher the pH (between pH 10-13), the higher the enzymatic monosaccharide release from the solid wheat fraction) (Figure 1b, d). For alkaline pretreatments an increase in pretreatment temperature from 100°C to 140°C only produced a weak, statistically insignificant increase in the enzymatic monosaccharide release (Table 1, Figure 1b, d). However, an increased pretreatment temperature in combination with increased pretreatment pH in the alkaline region resulted in improved enzymatic cellulose hydrolysis as manifested by a positive interaction between pH and temperature for the alkaline pretreatments (Table 1, Figure 1b). As discussed later, these yields were brought up significantly with increased enzyme dosage.

**Table 1:** Overview of the effects and interactions of pretreatment factors

Response	Fraction	Acid/alkali	pH	T	t	pH·T	pH·pH	T·t
Glucose	Solid	Acidic		x		x	x	-
	
	Liquid	Acidic	-					-

Xylose	Solid	Acidic	x	x		x	x	
	
	Liquid	Acidic	x					

Lignin	Solid	Acidic						x

Glucose	Solid	Alkaline	x			x	x	
	
	Liquid	Alkaline	x	x				x

Xylose	Solid	Alkaline	x		-		x	
	
	Liquid	Alkaline	x		x	x	x	

Lignin	Solid	Alkaline	x			x	x	

Significance of the pretreatment factors: pH, temperature (T), holding time (t) and their interactions on the responses enzymatic glucose release, enzymatic xylose release, and on lignin removal from the solid fraction. The significance estimates are based on multivariate linear regression of the responses (glucose and xylose releases) obtained after the standardized enzymatic hydrolysis treatment of samples obtained from the two sets of experimental designs, that is, acidic and alkaline.

x = *P *< 0.05; - = *P *< 0.10.

**Table 2:** Monosaccharide yields directly after pretreatment

Experiment	pH	T,°C	t, min	Glucose, g/kg	Xylose, g/kg
01	1.0	100	0	41	11

02	4.0	100	0	50	4

03	1.0	140	0	62	100

04	4.0	140	0	47	4

05	1.0	100	10	37	20

06	4.0	100	10	49	4

07	1.0	140	10	62	197

08	4.0	140	10	45	4

09	1.0	120	5	55	13

10	4.0	120	5	48	4

11	2.5	100	5	41	4

12	2.5	140	5	44	6

13	2.5	120	0	44	47

14	2.5	120	10	44	4

15 (CP)	2.5	120	5	39.0 ± 2.6	3.3 ± 0.6

16	10.0	100	0	35	3

17	13.0	100	0	99	68

18	10.0	140	0	56	5

19	13.0	140	0	130	126

20	10.0	100	10	38	4

21	13.0	100	10	121	113

22	10.0	140	10	54	4

23	13.0	140	10	140	142

24	10.0	120	5	45	3

25	13.0	120	5	122	126

26	11.5	100	5	61	6

27	11.5	140	5	80	9

28	11.5	120	0	52	6

29	11.5	120	10	49	6

30 (CP)	11.5	120	5	59.7 ± 3.5	6.7 ± 0.6

Monosaccharide yields in g/kg total original dry matter, obtained directly as a result of different pretreatment combinations (pH, temperature (T), and time (t)) in the experimental design sets of acid (experiments 1-15) and alkaline (experiments 16-30) pretreatments. CP = center point (n = 3) given as average ± SD. The experiments were performed in a completely randomized manner.

The absolute monosaccharide yields obtained from the pretreated solid wheat straw, given as g monosaccharide/kg dry matter, were generally significantly higher after the alkaline than after the acidic pretreatments. However, the release of xylose directly by the pretreatment was higher after the most extreme acidic pretreatment (pH 1, 140°C, 10 min) than after the corresponding most extreme alkaline pretreatment (pH 13, 140°C, 10 min), namely 197 versus 142 g/kg dry matter, whereas the direct release of glucose was higher after the most extreme alkaline pretreatment than after the most extreme acidic pretreatment, namely 140 versus 62 g/kg dry matter (Table 2). The relative increase in the enzymatic glucose release with increased pretreatment temperature was more pronounced, however, with the lowering of pH in the acidic pretreatments (that is, the multiple linear regression coefficient of the pH·T interaction was steeper (data not shown)) than that for the alkaline pretreatments (Figure 1a, b). Acidic pretreatments are known to release more monosaccharides/oligosaccharides directly during the pretreatment than alkaline pretreatments [17]. Apparently, this direct release of monosaccharides, which was not too high for glucose (Table 2), contributed to the low glucose and xylose yields obtained from the enzymatic treatments of the acid treated solid wheat straw fractions as compared to the yields from enzymatic hydrolysis of solids from the alkaline pretreatments.

For pretreatments at pH between 4 and 10, it has previously been shown that the releases of xylose and glucose are approximately constant and minimal [17]. Thus, the results signified that the choice of pH is crucial for obtaining the desired outcome of pretreatment at mild conditions. It is known that pretreatment at extreme pH values may produce elevated levels of potential inhibitors such as acetic acid, 5-hydroxymethyl-furfural, and 2-furfuraldehyde. We have recently found that pretreatment at pH 1 (with HCl) and at pH 13 (with NaOH) do produce elevated levels of these inhibitors as compared to what is obtained at less extreme pH values [17]. However, according to the available data on yeast fermentation, the inhibitor levels produced will not be significantly inhibitory for yeast fermentation [21-23], and any possible inhibitory effects will of course be further diminished if the liquid fraction is removed. Due to quick separation of liquids and solids, the amount of 5-hydroxymethyl-furfural and 2-furfuraldehyde produced in this work never exceeded 0.30 respectively 0.70 g/kg dry original matter wheat straw (only traces of these inhibitors were found at samples pretreated at pH 1). Regarding acetic acid, samples pretreated at pH 1 and pH 13 never exceeded 20 g/kg dry original matter wheat straw.

### Response interactions

**Figure 2:**
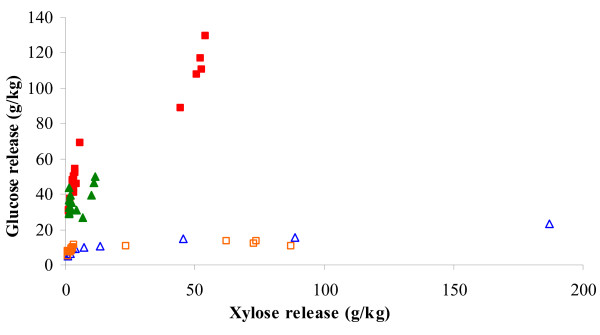
**Enzymatic glucose release as a response to total enzymatic xylose release**. Open blue triangles = liquid fractions for acidic, open red squares = liquid fractions for alkaline, filled green triangles = solid fractions for acidic, filled red squares = solid fractions for alkaline. All releases in g/kg originally dry matter.

Comparison of the interactions between the responses revealed the existence of several positive correlations between the enzymatically-released glucose and xylose in the different, pretreated wheat fractions (Figure 2). A plot of the glucose release versus the xylose release from the enzymatically hydrolyzed liquid fractions (Figure 2, open blue triangles and open red squares), showed that with a release of 10 g glucose/kg dry matter starting material, the xylose release might range from practically no release to about 80% of the theoretically maximal xylose release (Figure 2). That is, the enzymatic glucose solubilization only seemed slightly positively affected (if at all) by increased solubilization of hemicellulose during pretreatment. In contrast, the enzymatic release of glucose from the solid fractions seemed strongly positively affected by the simultaneous xylose release from the solid fraction, and this was the case after both acidic and alkaline pretreatments, but was particularly pronounced for the alkaline pretreatment (Figure 2, filled green triangles and filled red squares). For the solid wheat fraction, it thus seemed that the increased enzymatic xylose release increased the enzymatic glucose release, or vice versa.

**Figure 3:**
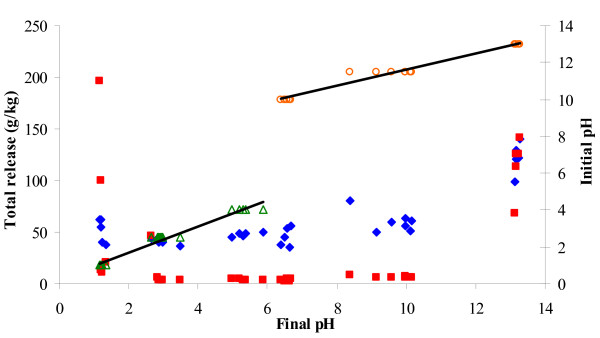
**Enzymatic release and initial pH as responses to final pH**. Total glucose (filled blue diamonds) and xylose (filled red squares) release as responses to final pH. Additionally, the initial pH is compared to the final pH by plotting initial pH of the acidic treatments (open green triangles) and the alkaline treatments (open red circles) as responses to the final pH. Black lines indicate fitted linear regression correlation curves (the regression coefficients, R^2^, exceeded 0.97 in both cases). Releases in g/kg original wheat straw dry matter.

To explain the correlated glucose-xylose release, the total glucose and the total xylose releases were plotted against the pH obtained after pretreatment (final pH) in the factorial designs (Figure 3). The enzymatic xylose release appeared to be unaffected by the final pH, when this pH ranged from pH 3-10, but was strongly affected at the highest and lowest pH values (Figure 3, filled red squares). That is, the more extreme the final pH levels after pretreatment, the more xylose was subsequently released enzymatically, but the exact xylose yields could not be predicted from the pretreatment pH alone (neither the initial nor the final pH) (Figure 3, filled red squares). In contrast, the enzymatic glucose release seemed less affected by the final pretreatment pH, although an increase in enzymatic release of glucose with both very low and especially with high final pH could be discerned (Figure 3, filled blue diamonds). The pH only changed slightly during pretreatment when more extreme initial pH values were used. However, pH was found to clearly change when less extreme initial pH values were used (Figure 3, open green triangles and open red circles). For example, at initial pretreatment pH values of 4 and 10, respectively, the final pH ended up being close to pH 6 (Figure 3, open green triangles and open red circles). We have previously found that the final pH after pretreatment of wheat straw may move toward approximately pH 6.3 [17]. The data presented here thus corroborated the trend that the final pH would move toward pH 6.0-6.3 when the pretreatment was initiated at less extreme pH values (Figure 3). Acetate, as well as other substances from the lignocellulose, is known to be released from xylan during pretreatment [7,14], but the mechanisms responsible for the pH nearing 6.3 cannot be concluded from any of the interactions described in this paper.

**Figure 4:**
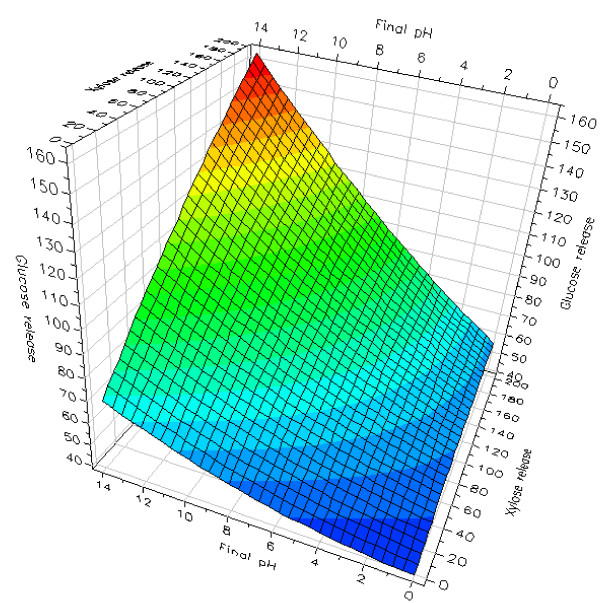
**Enzymatic glucose release as a response to xylose release and final pH**. Response surface model of total enzymatic glucose release as a response to total xylose release and the final pH. All releases in g/kg original wheat straw dry matter.

When the total enzymatic glucose release was modeled in response to the final pH (after pretreatment) and the total enzymatic xylose release, it became evident that increased enzymatic xylose release was positively correlated to the enzymatic glucose release, especially after alkaline pretreatment conditions (Figure 4). Hence, this model displayed a positive interaction between the final pH after pretreatment and the total enzymatic xylose release on the total enzymatic glucose release.

### Influence of holding time

The enzymatic xylose release from the liquid fraction from alkaline pretreatment was the only response that was significantly affected by the pretreatment holding time (Table 1); the effect was positive (that is, prolonged holding time increased the xylose release) (data not shown). However, even though not statistically significant, the enzymatic release of glucose and xylose from the liquid fractions from the acidic pretreatments also tended to increase with the increased holding time whereas the corresponding releases from the solid fractions decreased with increased holding time (data not shown). This pattern signified that both the alkaline and acidic hydrolysis of glycosidic bonds in cellulose and hemicellulose were influenced by holding time at the mild conditions.

### Lignin removal

**Figure 5:**
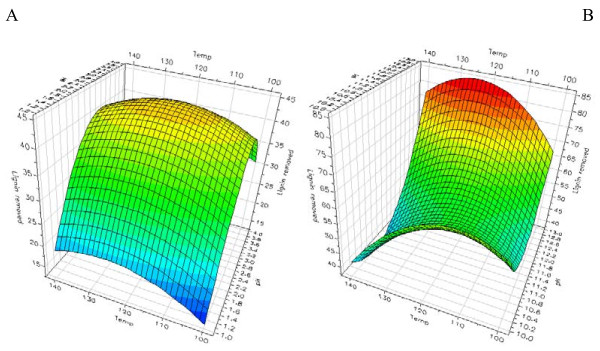
**Lignin removed from solid fractions as a response to pH and temperature**. **(a) **Acidic pretreatments; **(b) **alkaline pretreatments. Lignin removed measured in % (w/w) and temperature in°C.

The coefficients obtained by multiple linear regression analysis of the data showed that the pretreatment pH was the factor affecting the lignin removal the most (Table 1) (*P *= 0.0092). It is widely known, that alkaline treatment of lignocellulosic biomass affects the lignin removal [16,17]. However, to our knowledge there are no published data available on how the pretreatment pH quantitatively affects the lignin removal in combination with changes in pretreatment temperature and holding time. The response surface models resulting from the multiple linear regression analysis of the data showed that increase in pretreatment pH affected the lignin removal positively both in the acidic and alkaline region, but peaked at approximately pH 2.5 for the acidic pretreatments, and the effect of pH in the acid pretreatments therefore ended up as being a statistically insignificant factor, whereas it was statistically significant for the alkaline pretreatments (Table 1, Figure 5a). Moreover, the extent of lignin removal during the alkaline pretreatments was generally the double of that obtained by the acid pretreatments (Figure 5a, b). For pretreatment at 140°C the increase from pH 10 to 13 doubled the removal of lignin from 40% to 80% w/w (Figure 5b), and this interaction of pH and temperature was also statistically significant (Table 1). However, the multiple linear regression model within the chosen pH and temperature ranges, indicated that the maximal removal of lignin was at pH 13 and 125°C to 130°C (Figure 5b). Holding time during pretreatment also turned out to have a positive, but statistically insignificant influence on the lignin removal: hence the extent of lignin removal increased by approximately 10% (weight/weight dry matter) when increasing the holding time from 0 to 5 min and again when increasing from 5 to 10 min (data not shown).

**Figure 6:**
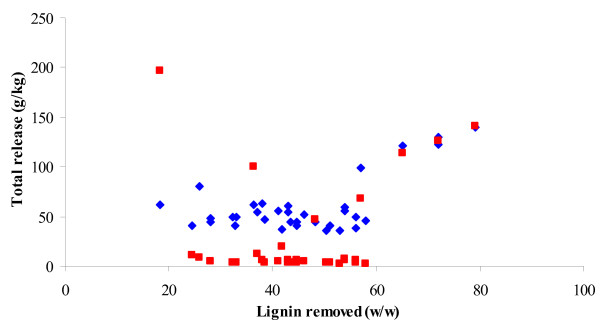
**Enzymatic glucose and xylose release as responses to lignin removed**. Total glucose (filled blue diamonds) and xylose (filled red squares) release in g/kg originally dry matter.

The release of lignin and xylan from lignocellulosic biomass is known to affect the cellulose digestibility positively for corn stover [16]. However, a plot of the enzymatic monosaccharide yields obtained on solid wheat straw in response to extent of lignin removal showed that the enzymatic releases of both glucose and xylose increased linearly with lignin removal only when the lignin removed was above 60% by weight of the original level in the wheat straw biomass (Figure 6). The data with > 60% lignin removal were all from the alkaline pretreatment. The data points for the very low extents of lignin removal obtained after acidic pretreatments (≤ 20% w/w lignin removed, Figure 6) make the postulated influence of any lignin removal on the subsequent enzymatic release of monosaccharides uncertain. Lignin, including lignin monomers liberated during biomass pretreatment, may inhibit the hydrolytic enzymes and the fermenting microorganisms; hence, the removal of lignin is in any case advantageous for the further processing of the biomass for biofuel production.

### Effect of enzyme dosage on enzymatic hydrolysis of pretreated wheat straw

**Figure 7:**
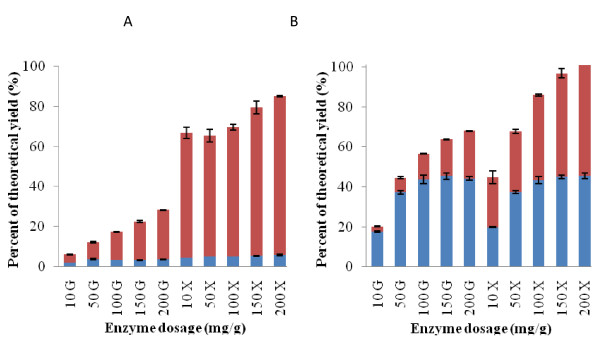
**Monosaccharide yield response to enzyme dosage**. Enzyme catalyzed release of glucose (G) and xylose (X) from solid (blue) and liquid fractions (red) when pretreated at A: pH 1, B: pH 13. The x axes show added enzyme concentration in mg enzyme protein (EP)/g dry weight biomass (benchmark dosage used for assessing the effects of pretreatment factors was 10 mg EP/g). Releases are given in percentage theoretical, maximal yield.

In order to make sure that any significant and interactive effects of pretreatment factors could be discerned after enzymatic hydrolysis on both the liquid and solid wheat straw fractions resulting after pretreatment, a very low enzyme dosage, 10 mg enzyme protein/g (original untreated) wheat straw dry matter, was deliberately chosen as a benchmark enzyme treatment in the experimentally designed experiments. As briefly discussed above, only low amounts of glucose and xylose were released at this low enzyme dosage. When increasing the enzyme dosage in the hydrolysis of the biomass pretreated at 140°C at pH 1 for 10 min, the yields of glucose did not exceed 5% of the theoretical maximal release (Figure 7a). The glucose yield from the liquid part increased significantly with increased enzyme dosage, but the final total enzymatic glucose yield at the highest enzyme dosage of 200 mg EP/g substrate dry matter never gave yields beyond approximately 25% of the theoretical maximum (Figure 7a). This indicated that the short (10 min), low temperature pretreatment (140°C), albeit run at pH 1, was not sufficiently effective to prepare the cellulose for enzymatic attack. Regarding the degradation of the hemicelluloses (that is, mainly xylan), the high release of xylose in the liquid fractions (up to approximately 80% of the theoretical maximum) (Figure 7a) confirmed the significant impact of acid pretreatment on hemicellulose solubilization from lignocellulosic biomass [14].

In contrast, the extreme alkaline pretreatment (that is, pH 13, 140°C, 10 min) prepared the material well for enzymatic liberation of glucose and xylose as the enzyme catalyzed monosaccharide release increased significantly with increased enzyme dosage. This effect was seen with both the solid and the liquid fractions (Figure 7b). The total release of glucose reached 65% to 70% of the theoretical maximum when the enzyme dosage was increased to 150-200 mg/g whereas the enzyme catalyzed xylose liberation from the solid and liquid fractions reached 100% at an enzyme dosage of 100-150 mg/g (Figure 7b). When considering that the most extreme alkaline pretreatment (that is, pH 13, 140°C, 10 min) also induced release of approximately 75% to 78% of the lignin (Figure 5b) the mass balance showed that approximately 30% to 35% of the available glucose was not liberated and that 22% to 25% of the lignin was not solubilized; however, the enzymatic treatment was conducted as a 24 h enzymatic hydrolysis treatment and the enzyme catalyzed glucose liberation may increase with extended reaction time.

## Conclusions

In the present work we have shown that alkaline pretreatment pH is better for preparing for enzymatic release of both xylose and glucose than acid pretreatment pH when treatment conditions are mild (T ≤ 140°C, 10 min treatment). Furthermore, at mild conditions (T ≤ 140°C), pH was the most significant factor influencing the removal of lignin from the wheat straw biomass and the subsequent enzymatic release of monosaccharides. The pretreatment pH also had a significant interaction effect with the pretreatment temperature on the subsequent enzymatic release of glucose from the wheat straw biomass (but not of xylose after alkaline pretreatment). In general, alkaline pretreatment appeared to make the solid wheat straw biomass more amenable to enzymatic attack. Acidic pretreatment catalyzed the solubilization of hemicellulosic biomass prior to enzymatic hydrolysis, thus liberating a crucial amount of possible fermentable C5 monosaccharides directly into the liquid fraction; this solubilization may cause losses if only the solid fraction is further processed. It is certain that the pretreatment factor interactions, including the influence on lignin solubilization for enzymatic digestibility, need to be better understood to improve future pretreatment strategies for more cost effective lignocellulose processing.

## Methods

### Substrate preparation and composition

Wheat straw grown in Grumløse (southern Zealand, Denmark) was obtained from The Danish Cooperative Farm Supply, Bårse, Denmark. The substrate was sorted manually to contain only stems and the material was then ground repeatedly by use of a cutting mill (Retsch SM 2000, Haan, Germany) and separated into different particle sizes by steel sieves (Endecotts, London, UK); the straw used in the present study had particle sizes of 700-1,000 μm [12]. Based on compositional analysis of structural carbohydrates and lignin by means of the National Renewable Energy Laboratory (NREL) method [24] the composition of the ground wheat straw defining the maximal theoretical monosaccharide yields, was 536 g glucose/kg, 220 g xylose/kg and 162 g lignin/kg with only minor amounts of arabinose and galactose and 1 g ash/kg (kg indicates per kg dry matter). Monosaccharides were analyzed by high performance anion exchange chromatography (HPAEC) as outlined below. The lignin analyses performed on the solid wheat fractions after different pretreatments were also performed by the standardized NREL method [24].

### Hydrothermal treatment of wheat straw

The hydrothermal treatment was performed in a custom-built looped progressive cavity pump reactor (ADP 0.8 × 3 by Allweiler, AxFlow A/S, Smørum, Denmark) with a loop consisting of a stainless steel tube (26.9 × 2.6 by Sanistål A/S, Copenhagen, Denmark) with a total volume of 0.6 l [25]. Ground wheat straw was mixed with water to give a dry matter content of 2% dry matter (w/w). Catalyst was added to adjust the initial pH of the straw slurry (initial pH) according to the experimental plan, and the slurry was poured into the reactor through a stainless steel funnel. When the biomass was added, the inlet was sealed, the flow rate was set to 1 l/min and the tube was heated from 25°C to 140°C at 5.4°C per min. After pretreatment, the loop was cooled from 140°C to 80°C by 11°C per min prior to emptying the reactor, and the biomass was separated while still warm to minimize solubilized xylan fractions to redeposit onto the solid wheat straw fraction [19]. The separation of the solid and liquid fraction took place in glass filter crucibles and the pH after pretreatment was measured (final pH). The total amount of solid dry matter remaining after pretreatment was measured prior to measuring the amount of acid insoluble lignin remaining in the solid fraction. Afterwards, the solid and the liquid fractions were enzymatically treated separately (see below).

### Enzymatic hydrolysis

Enzymatic hydrolysis reactions were carried out by treatment with a novel commercial enzyme blend Cellic C-Tec derived from *Trichoderma reesei *(Novozymes A/S, Bagsværd, Denmark). Apart from the cellulolytic enzyme base from *T. reesei *containing at least the two main cellobiohydrolases EC 3.2.1.91 (Cel6A and Cel7A), five different endo-1,4-β-glucanases EC 3.2.1.4 (Cel7B, Cel5A, Cel12A, Cel61A, and Cel45A), β-glucosidase EC 3.2.1.21, and a β-xylosidase [26,27] the Cellic C-Tec also contains additional β-glucosidase and particular glycoside hydrolase family 61 hydrolysis-boosting proteins [28]. The Cellic C-Tec preparation contained 175 mg protein/ml, 63.8 filter paper units (FPU)/ml and 868 cellobiase units (CBU)/ml; the FPU were determined according to the standardized FPU determination procedure provided by NREL [29], and CBU were determined by the assay described by Ghose [30], respectively. The enzymatic hydrolysis reactions of the solid, pretreated wheat samples were performed as described previously [12,17]: Briefly, the reactions took place at 50°C, pH 5.0 (using 0.1 M sodium acetate buffer with 0.02% by weight of sodium azide) with 2% dry matter substrate (w/w) in Eppendorf tubes (Eppendorf, Hamburg, Germany) spun at 750 rpm; the liquid filtrate samples were diluted 100 times prior to enzymatic hydrolysis. The Cellic C-Tec preparation was dosed based on mg enzyme protein/g substrate dry matter (from 10-200 mg enzyme protein/g dry matter). The enzymatic hydrolyses were stopped after 24 h by heating of the samples at 100°C for 10 min. The samples were then cooled to room temperature, centrifuged at 10,000 rpm for 10 min and the levels of glucose and xylose liberated were determined by HPAEC using a Dionex BioLC system equipped with a Dionex CarboPac PA1 analytical column (Dionex, Sunnyvale, CA, USA) and an electrochemical detector used in the pulsed amperiometric detection mode principally as described previously [31]; in brief, the monosaccharides were separated via a two-step isocratic procedure using 25 mM NaOH for 5 min, then 10 mM NaOH for 13 min. Each run included cleaning of the column with 500 mM NaOH for 7 min, and re-equilibration with 25 mM NaOH for 5 min.

### Experimental design and statistics

The statistical software MODDE 7.0 (Umetrics AB, Umeå, Sweden) was used as an aid for designing the Central Composite Face experimental designs and for analyzing the data by multiple linear regression. The pretreatment temperature, pH (initial pH), and the holding time were varied according to a 15-point central composite design with the center point repeated 3 times. Correlation coefficients of glucose versus xylose releases were determined by linear regression analysis. The statistical significance of the correlations was evaluated by the dose-response *F *test [32].

### Calculations

The yields were calculated as g liberated of glucose and xylose, respectively, per kg of dry weight of non-pretreated wheat straw (m_DM(before pretreatment)_, w/w) as described previously [17]. The monosaccharide yields from enzymatically hydrolyzed solid fractions were calculated as:

where d is the dilution factor, C the concentration of monosaccharide in g/l as obtained by HPAEC. V_liquid in hydrolysis _is the volume made up by liquid in the hydrolysis to account for solids taking up volume, and m_DM(hydrolysis) _in kg is the dry weight of solid being hydrolyzed to calculate the yield of monosaccharide released from hydrolysis per kg water insoluble solids. To present the release of monosaccharide as g per kg original dry weight non-pretreated wheat straw, the yield was multiplied by the factor of solid biomass after pretreatment divided by solid biomass before pretreatment (m_DM(after pretreatment)_/m_DM(before pretreatment)_). The monosaccharide yields from enzymatically hydrolyzed liquid fractions were calculated as:

where V_liquid after pretreatment _is the volume of liquid measured after each pretreatment.

## Competing interests

The authors declare that they have no competing interests.

## Authors' contributions

All authors contributed intellectually via scientific discussions during the work and have read and approved the final manuscript. MP designed the study, executed the experimental work and drafted the manuscript. KSJ commented on the manuscript. AM contributed to the design of the study, the experimental design, the statistical data analysis and helped write the paper.
